# Three-month outcomes of faricimab loading therapy for wet age-related macular degeneration in Japan

**DOI:** 10.1038/s41598-023-35759-4

**Published:** 2023-05-30

**Authors:** Ryo Mukai, Keiko Kataoka, Koji Tanaka, Yasunori Miyara, Ichiro Maruko, Makiko Nakayama, Yuto Watanabe, Akiko Yamamoto, Yu Wakatsuki, Hajime Onoe, Sorako Wakugawa, Nobuhiro Terao, Taiji Hasegawa, Nozomu Hashiya, Moeko Kawai, Ruka Maruko, Kanako Itagaki, Jyunichiro Honjo, Annabelle A. Okada, Ryusaburo Mori, Hideki Koizumi, Tomohiro Iida, Tetsuju Sekiryu

**Affiliations:** 1 Department of Ophthalmology, Fukushima Medical University, 1 Hikarigaoka-Cho, Fukushima, 960-1295 Japan; 2 Department of Ophthalmology, Kyorin University School of Medicine, Tokyo, Japan; 3 Department of Ophthalmology, Nihon University School of Medicine, Tokyo, Japan; 4 Department of Ophthalmology, Graduate School of Medicine, University of the Ryukyus, Okinawa, Japan; 5 Department of Ophthalmology, Tokyo Women’s Medical University, Tokyo, Japan

**Keywords:** Diseases, Eye diseases

## Abstract

This multicenter study aimed to assess the short-term effectiveness and safety of faricimab in treatment-naïve patients with wet age-related macular degeneration (wAMD) in Japan. We retrospectively reviewed 63 eyes of 61 patients with wAMD, including types 1, 2, and 3 macular neovascularization as well as polypoidal choroidal vasculopathy (PCV). Patients received three consecutive monthly intravitreal injections of faricimab as loading therapy. Over these 3 months, visual acuity improved gradually compared to baseline. Moreover, the central foveal thickness decreased significantly at 1, 2, and 3 months compared to baseline (*p* < 0.0001). At 3 months after initiation of faricimab therapy, a dry macula (defined as absence of intraretinal or subretinal fluid) was achieved in 82% of the eyes. Complete regression of polypoidal lesions was observed in 52% of eyes with PCV. Subfoveal choroidal thickness also decreased significantly at 1, 2, and 3 months compared to baseline (*p* < 0.0001). Although retinal pigment epithelium tears developed in two eyes, there were no other ocular or systemic complications observed during the 3 months of loading therapy. In conclusion, loading therapy using faricimab resulted in improved visual acuity and retinal morphology in Japanese patients with wAMD without particular safety issues.

## Introduction

Worldwide, age-related macular degeneration (AMD) is a leading cause of central vision loss in the elderly population^1–3^. In the pathologic phase of macular neovascularisation (MNV) in wet AMD (wAMD), angiopoietin-2 (Ang-2) expression increases in the vascular endothelium; this competitively inhibits angiopoietin-1 (Ang-1) from binding tyrosine kinase immunoglobulin-like receptors (Tie 2) on the surface of vascular endothelial cells, thus preventing Ang-1/Tie 2 signalling and resulting in capillary inflammation or pericyte loss^4^. In patients with wAMD, Ang-2 expression is upregulated in the retina and vitreous^5^.

Vascular endothelial growth factor (VEGF) inhibitors are the main treatment option for patients with wAMD; there are several commercially available anti-VEGF agents, including bevacizumab (off-label), ranibizumab, aflibercept, and brolucizumab^6,7^. Intensive research has been conducted on optimal treatment protocols based on these drugs for wAMD^8^. In 2022, the first humanized, bispecific IgG monoclonal antibody, faricimab, which inhibits both VEGF-A and Ang-2, was approved after two phase 3 trials (TENAYA and LUCERNE). It demonstrated the visual benefits of treatment with faricimab given at up to 16-week intervals in patients with wAMD^9^. This study aimed to evaluate the safety and efficacy of faricimab treatment for patients with wAMD in real-world settings.

## Results

**Figure 1:**
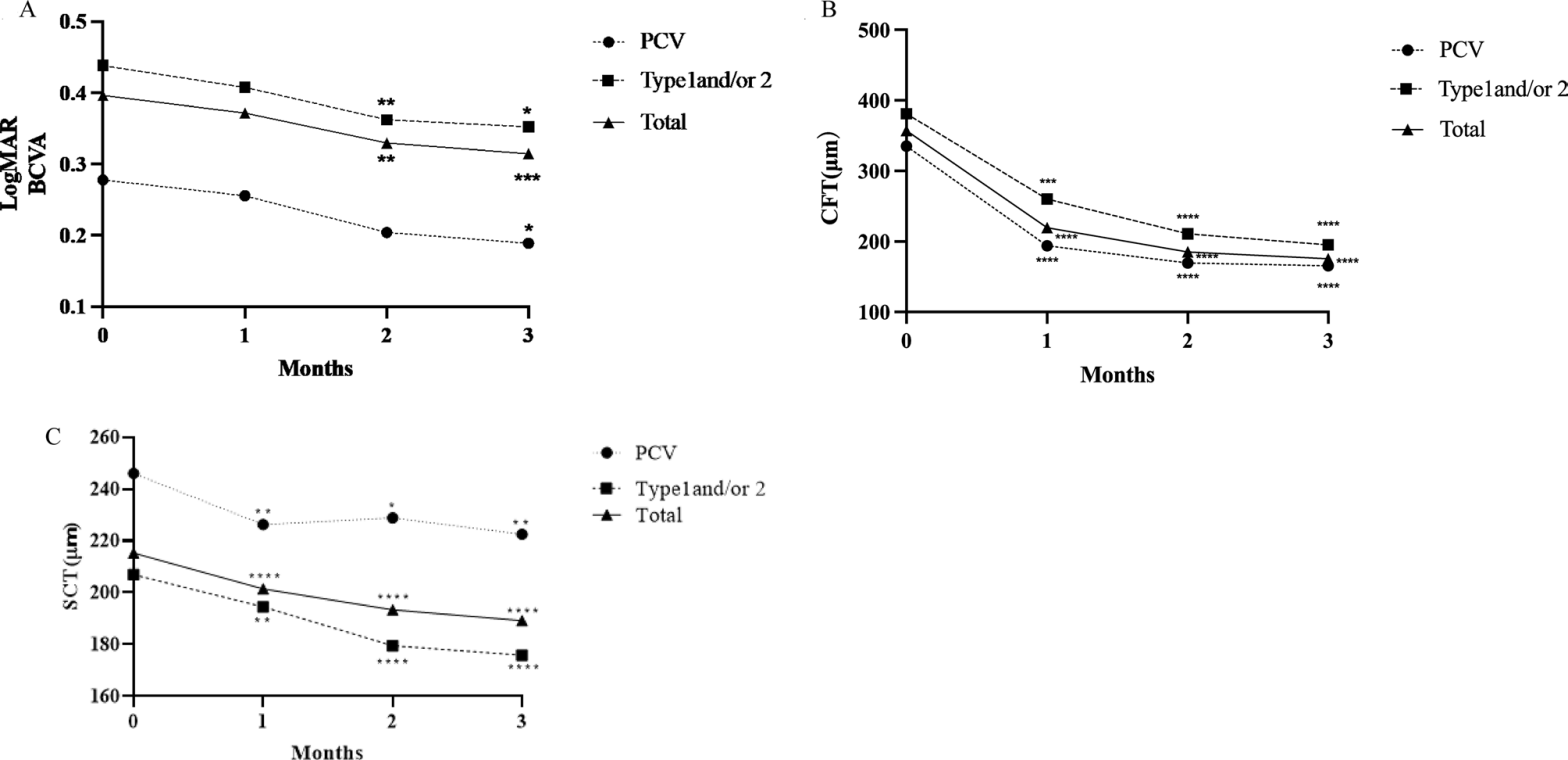
(**A**) Changes in best-corrected visual acuity in 62 eyes treated with 3 monthly faricimab injections. *p < 0.05, **p < 0.01, ***p < 0.001. (**B**) Changes in average central foveal thickness (CFT) in 62 eyes treated with 3 monthly faricimab injections. ***p < 0.001, ****p < 0.0001. (**C**) Changes in average subfoveal choroidal thickness (SCT) in 62 eyes treated with 3 monthly faricimab injections. *p < 0.05, **p < 0.01, ****p < 0.0001.

**Figure 2:**
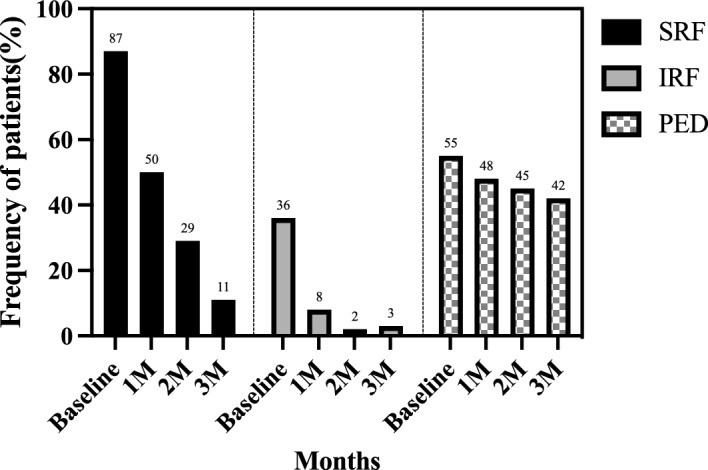
Presence of subretinal fluid (SRF), intraretinal fluid (IRF) and pigment epithelial detachment (PED) at baseline, 1, 2, and 3 months after initiation of faricimab.

**Figure 3:**
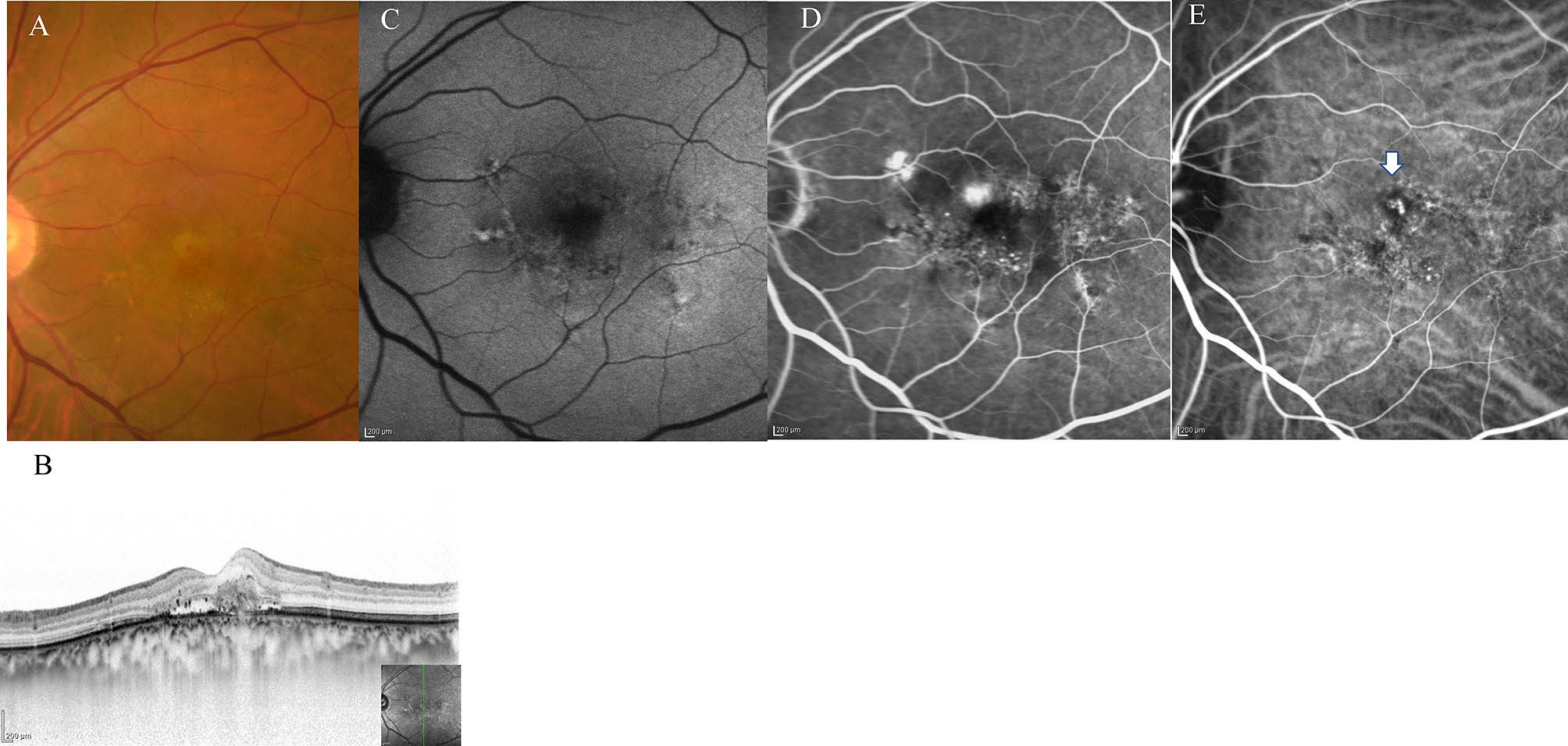
*Case 1* A 75-year-old male patient with PCV at baseline. (**A**) Fundus photography revealed pigment epithelial detachment (PED) with fibrin at the fovea. (**B**) Optical coherence tomography (OCT) revealed PED with fibrin and subretinal fluid. (**C**) Fundus autofluorescence imaging revealed a blockage of the PED. (**D**) Late-phase fluorescein angiography revealed leakage from polypoidal lesions at the macula. (**E**) Middle-phase indocyanine green angiography identified polypoidal lesions at the fovea (arrowhead) with a branching neovascular network.

**Figure 4:**
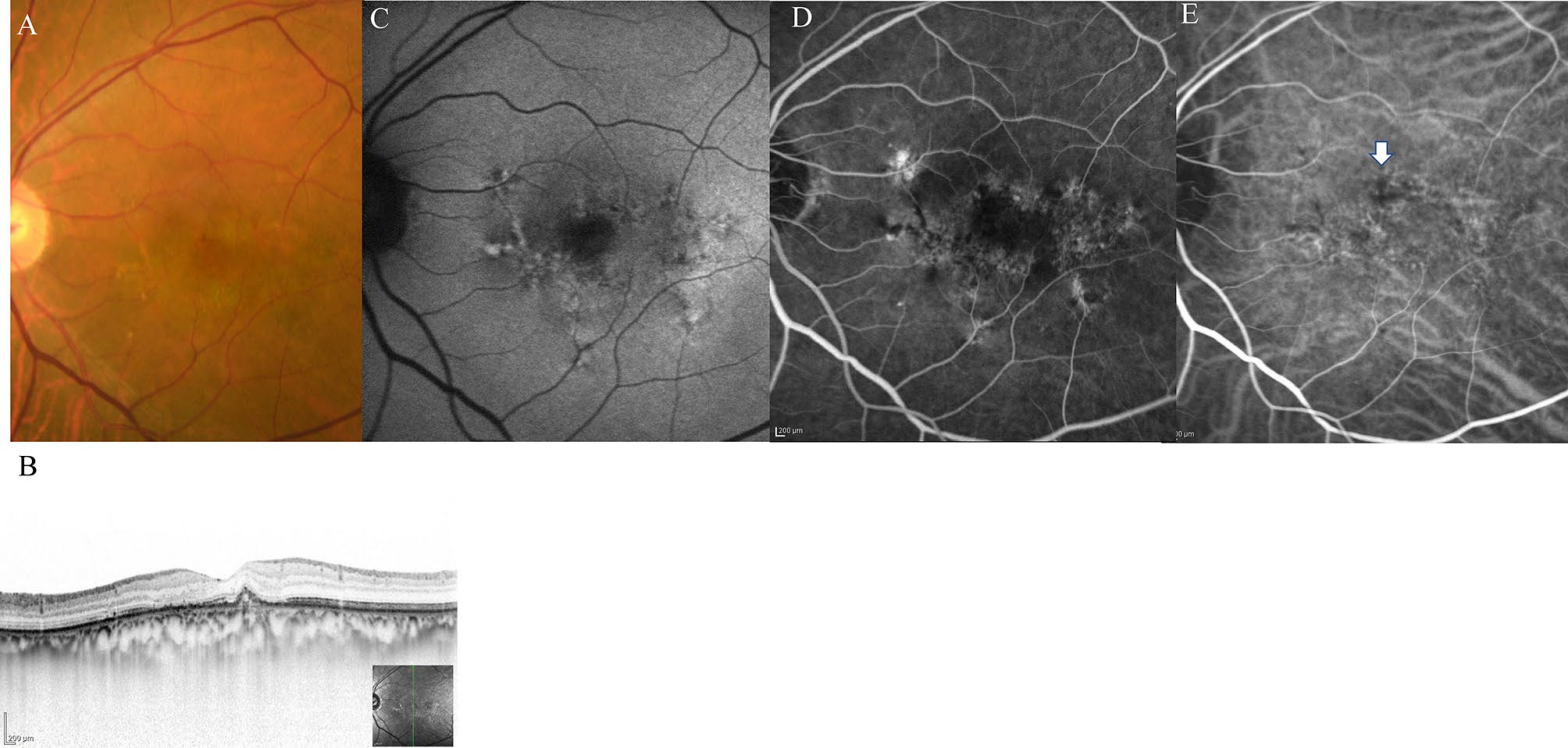
The same 75-year-old male patient as shown in Fig. 1, here at 3 months after loading therapy with faricimab. (**A**) Fundus photography shows partial regression of pigment epithelial detachment (PED) as well as complete fibrin absorption at the fovea. (**B**) Optical coherence tomography (OCT) revealed regressed PED and absorption of serous retinal detachment. (**C**) Fundus autofluorescence imaging revealed no enlargement of the patchy atrophy. (**D**) Late-phase fluorescein angiography detected no leakage from a polypoidal lesion at the fovea. (**E**) Middle-phase indocyanine green angiography identified no polypoidal lesions at the fovea.

**Figure 5:**
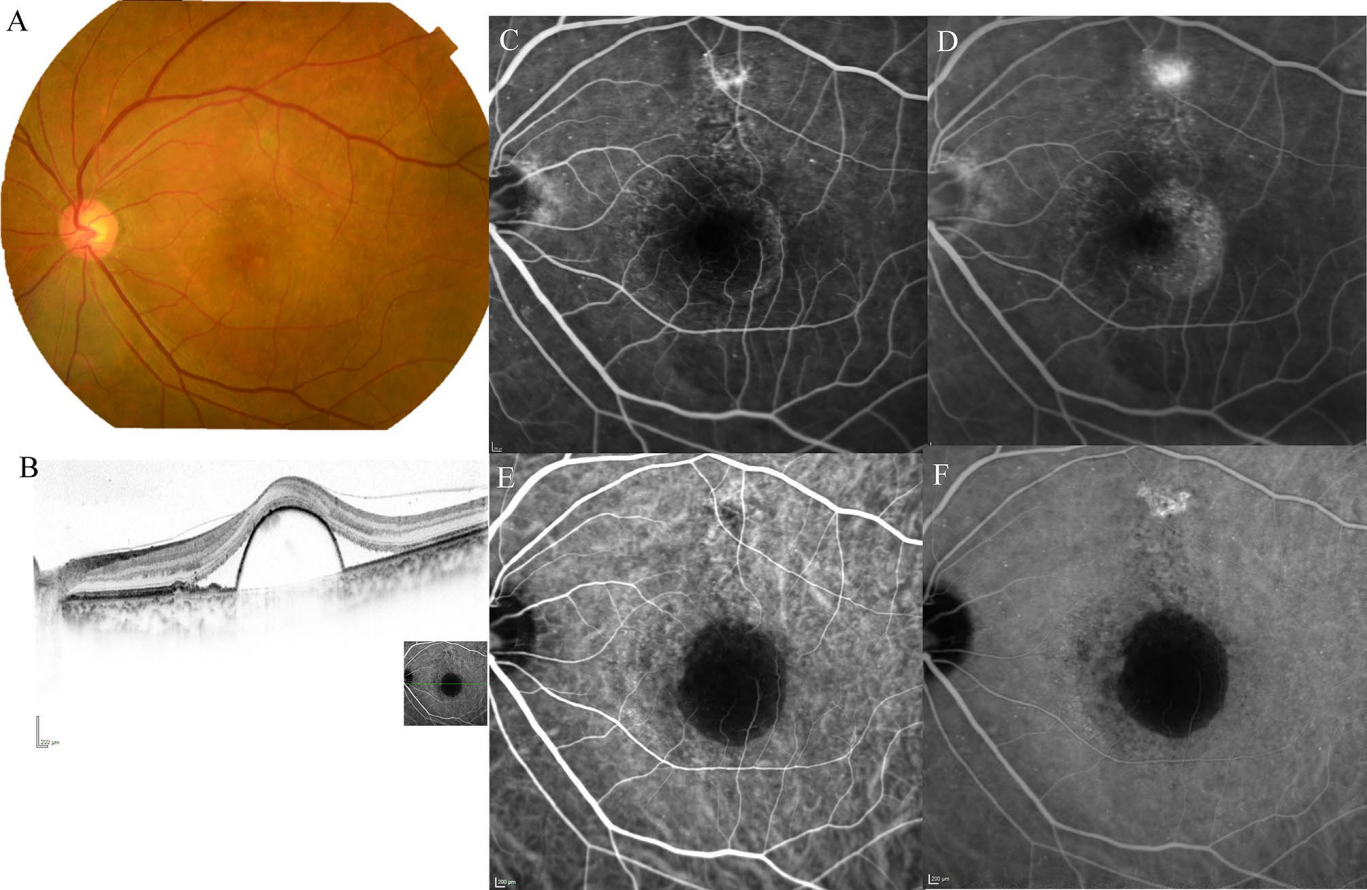
*Case 2* A 63-year-old male patient with type 1 macular neovascularisation (MNV) at baseline. (**A**) Fundus photography shows a pigment epithelial detachment (PED) with subretinal fluid (SRF) at the fovea. (**B**) Optical coherence tomography (OCT) revealed a PED with SRF and a shallow PED at the nasal side of the large PED. (**C**, **D**) Early and late-phase fluorescein angiography detected a type 1 MNV. (**E**, **F**) Early and late-phase indocyanine green angiography identified abnormal MNV at the nasal edge of the large PED.

**Figure 6:**
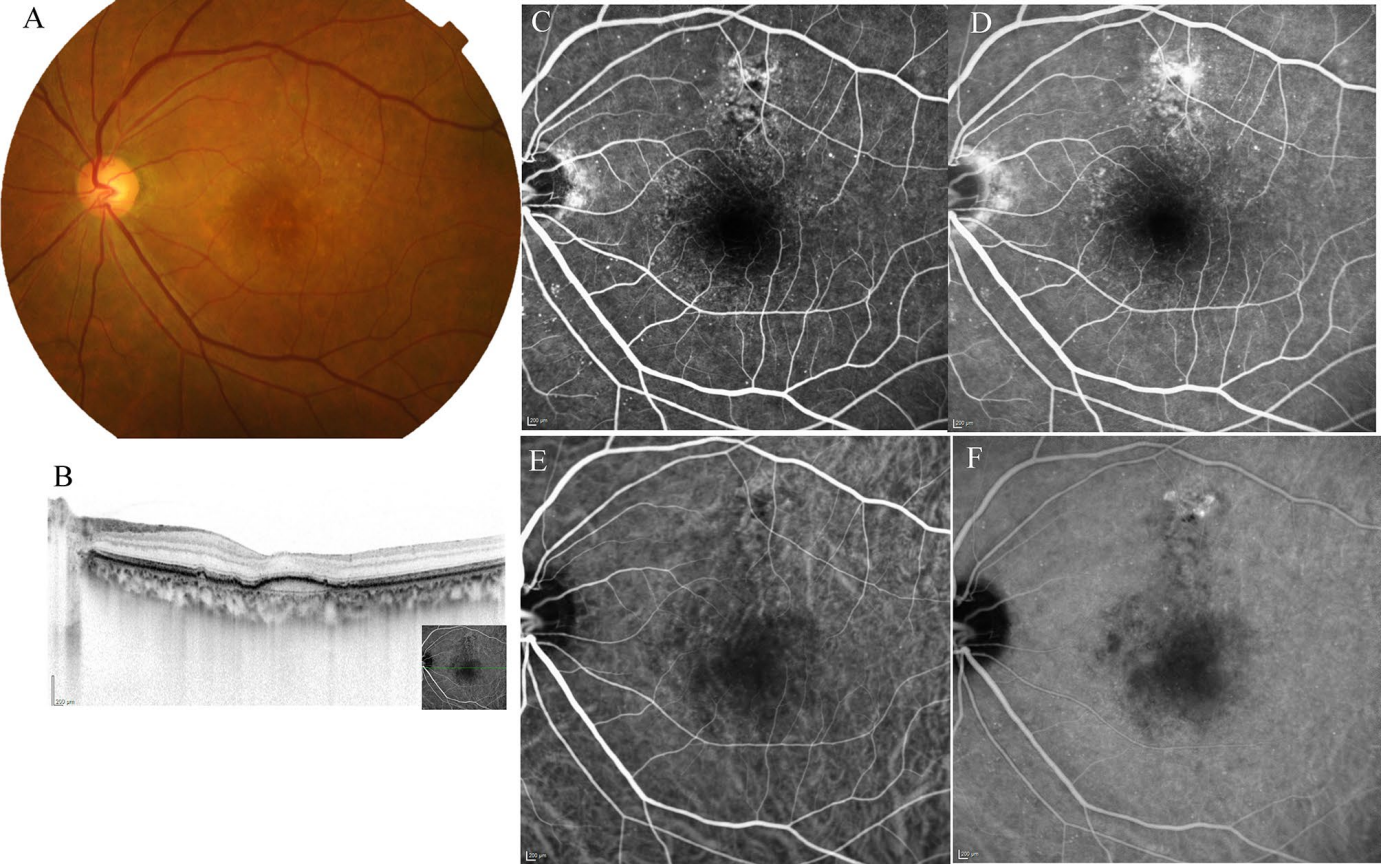
The same 63-year-old male patient as shown in Fig. 6, here at 3 months after initiation of faricimab therapy. (**A**) Fundus photography shows absorption of the subretinal fluid (SRF), and the pigment epithelial detachment (PED) was well absorbed. (**B**) Optical coherence tomography (OCT) revealed absorption of SRF, and a drastic decrease in the height of PED. (**C**, **D**) Early and late-phase fluorescein angiography detected no leakage from MNV. (**E**) Early and late-phase indocyanine green angiography identified no blockade of the large PED.

**Figure 7:**
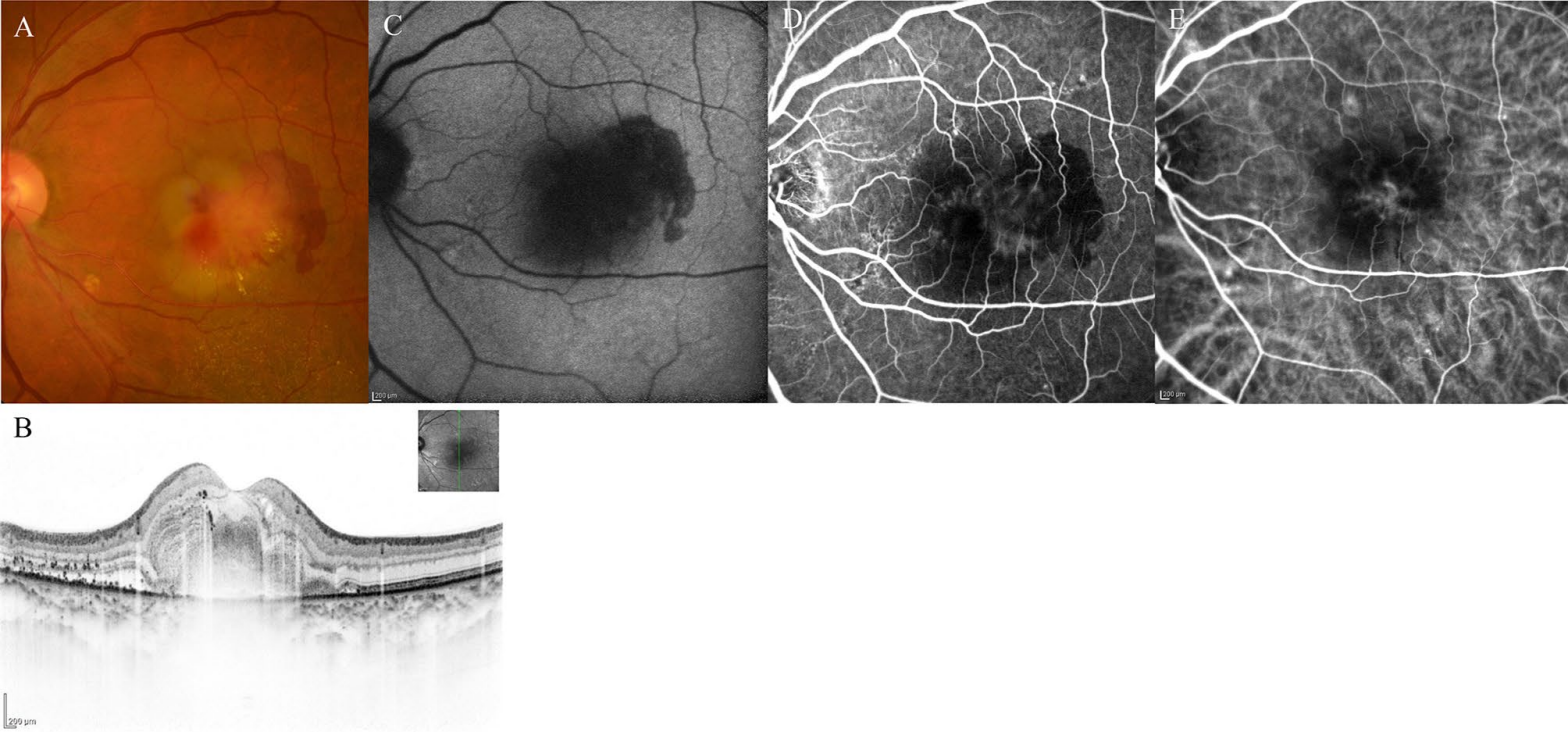
A 68-year-old male patient with type 2 macular neovascularisation (MNV) at baseline. (**A**) Fundus photography shows yellowish MNV surrounded by retinal haemorrhage with fibrin at the fovea. (**B**) Optical coherence tomography (OCT) revealed a subretinal mass surrounded by haemorrhage and fibrin with subretinal fluid. (**C**) Fundus autofluorescence imaging revealed a hemispherical blockade due to the subretinal haemorrhage. (**D**) Middle-phase fluorescein angiography detected MNV; however, haemorrhage caused a blockade. (**E**) Middle-phase indocyanine green angiography identified abnormal choroidal neovascularisation at the fovea.

**Figure 8:**
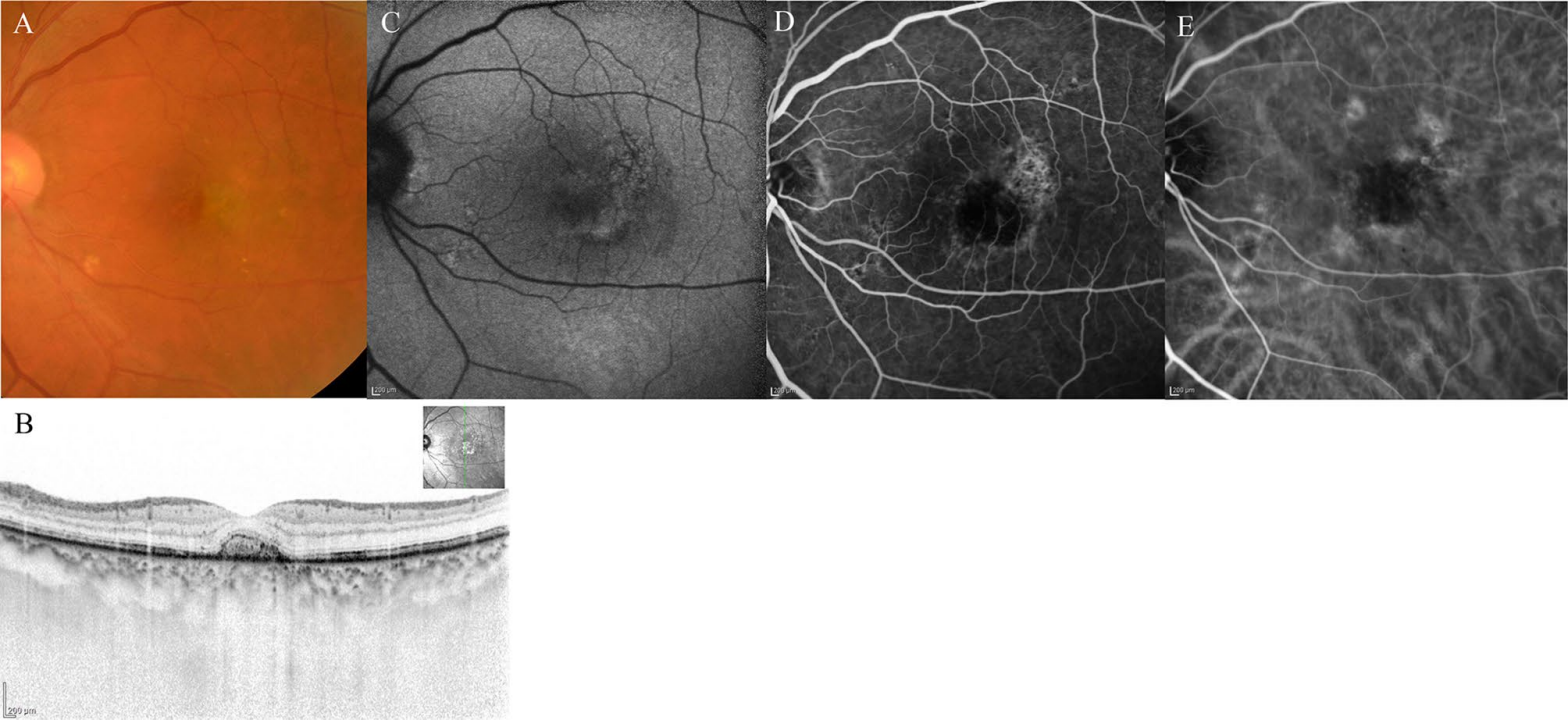
The same 68-year-old male patient as shown in Fig. 7, here at 3 months after initiation of faricimab therapy. (**A**) Fundus photography shows absorption of the haemorrhage, fibrin, and yellowish mass. (**B**) Optical coherence tomography (OCT) revealed absorption of fibrin and haemorrhage, serous retinal detachment, and a small mass retained in the subretinal region. (**C**) Fundus autofluorescence imaging revealed very slight fine granular atrophy. (**D**) Late-phase fluorescein angiography detected no leakage from MNV. (**E**) Middle-phase indocyanine green angiography identified no abnormal vasculature.

**Figure 9:**
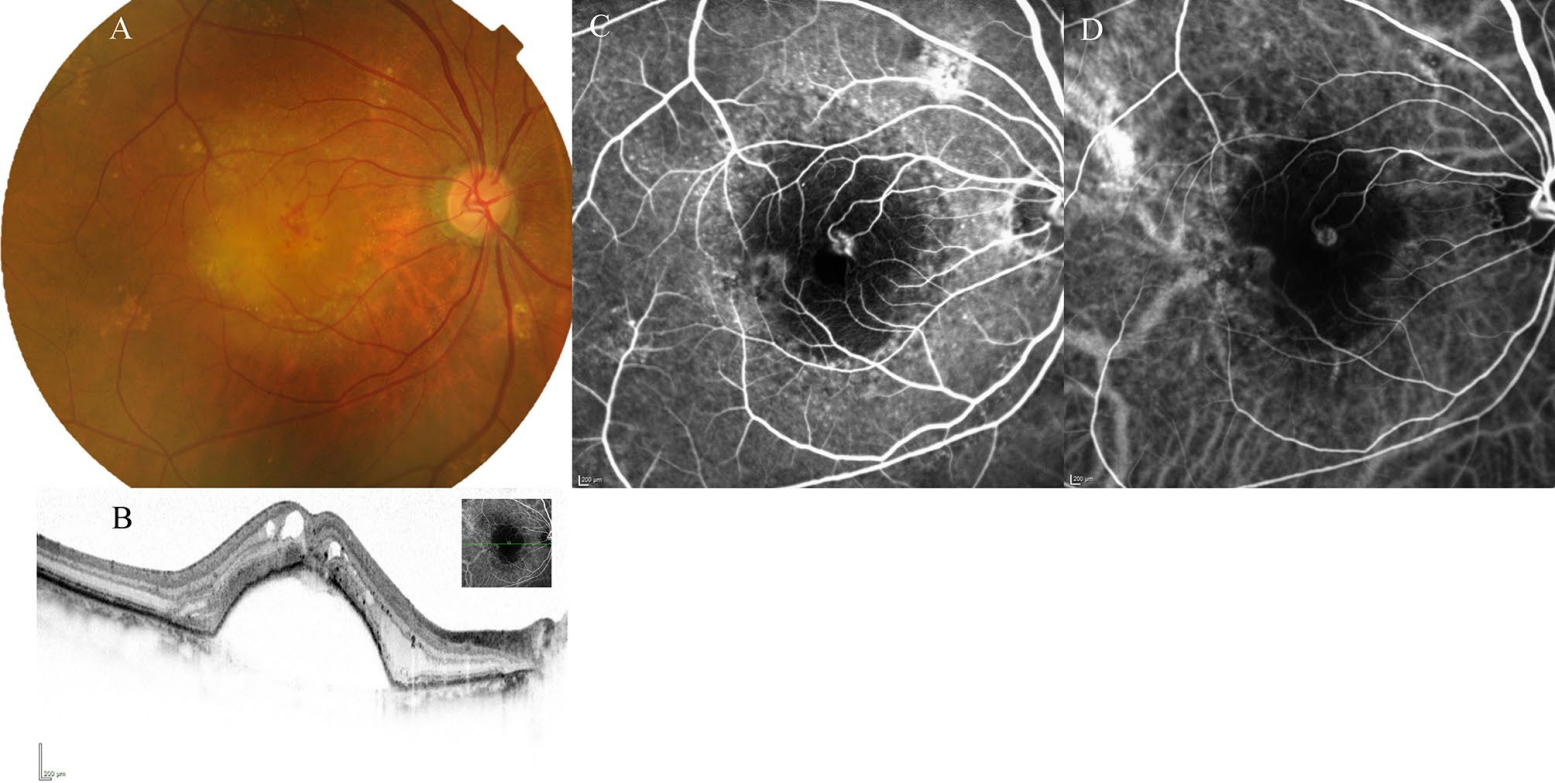
*Case 4* A 78-year-old female patient with type 3 macular neovascularisation (MNV) at baseline. (**A**) Fundus photography revealed a pigment epithelial detachment (PED) with intra retinal fluid (IRF) and intraretinal haemorrhage at the fovea. (**B**) Optical coherence tomography (OCT) revealed the large PED with IRF. (**C**) Early-phase fluorescein angiography detected dye leakage from a type 3 MNV. (**D**) Early-phase indocyanine green angiography also identified the type 3 neovascularisation.

**Figure 10:**
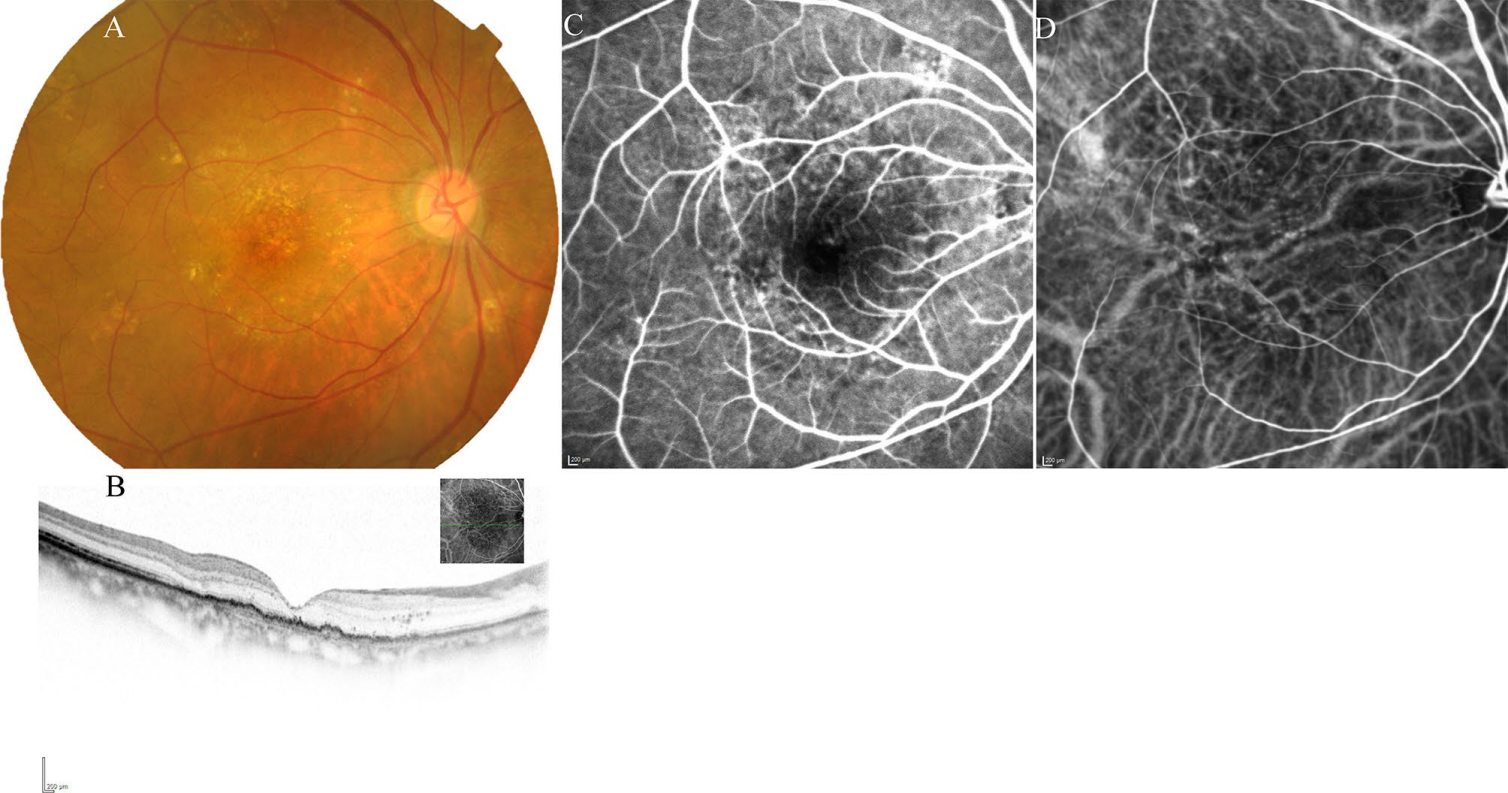
The same 78-year-old female patient as shown in Fig. 9, here at 3 months after initiation of faricimab therapy. (**A**) Fundus photography shows absorption of the intraretinal fluid (IRF) and disappearance of intra retinal haemorrhage. The pigment epithelial detachment (PED) was absorbed. (**B**) Optical coherence tomography (OCT) revealed absorption of the IRF, and the PED was almost completely regressed. (**C**, **D**) Early-phase fluorescein angiography detected no leakage from the type 3 macular neovascularisation (MNV). (**E**) Early-phase indocyanine green angiography identified no type 3 MNV.

**Figure 11:**
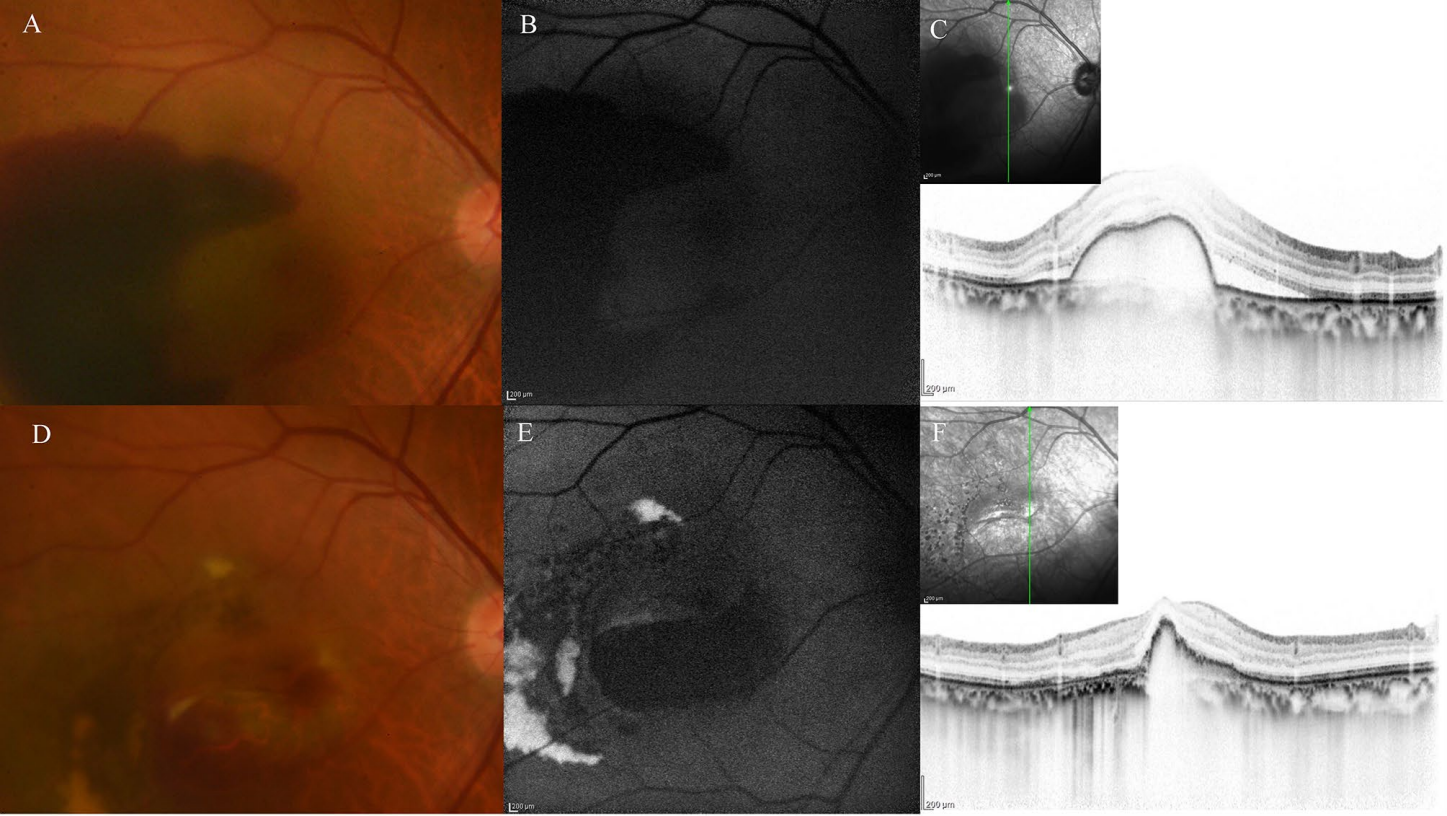
A case of retinal pigment epithelial (RPE) tear in 77-year-old male patient with polypoidal choroidal vasculopathy. (**A**) Fundus photography revealed a haemorrhagic pigment epithelial detachment (PED) with subretinal fluid (SRF) and subretinal haemorrhage at the fovea. (**B**) Fundus autofluorescence (FAF) revealed no RPE tear at baseline (**C**) Optical coherence tomography (OCT) revealed the large haemorrhagic PED with SRF. (**D**) Fundus photography at 1 months after the third injection of faricimab revealed that RPE tear developed at the inferior of the PED. (**E**) FAF identified hemispherical RPE defect at the inferior part of the PED. (**F**) OCT revealed RPE defect at the inferior part of the pre-existing PED.SRF was completely absorbed. Regardless of the RPE tear development, visual acuity improved from 20/125 to 20/40.

The background characteristics of the participating patients with wAMD are presented in Table 1. Among the 62 included eyes, there were 32 (52%) eyes with type 1 and/or type 2 MNV (24 eyes with type 1 MNV, 4 eyes with type 2 MNV, and 4 eyes with both), 22 (35%) eyes with polypoidal choroidal vasculopathy (PCV), and 8 (13%) eyes with type 3 MNV. The means best corrected visual acuity (BCVA) at baseline 1, 2, and 3 months after initiation of faricimab were 0.40 ± 0.42, 0.37 ± 0.47, 0.33 ± 0.42, and 0.32 ± 0.43, respectively. The BCVA significantly improved at 2 and 3 months after the initiation of faricimab administration *(p* < 0.01, and *p* < 0.01, respectively; Fig. 1-A). The mean central foveal thickness (CFT) values at baseline, 1, 2, and 3 months after initiation of faricimab were 357 ± 165 μm, 219 ± 140 μm, 185 ± 94 μm, and 175 ± 91 μm, respectively. CFT was significantly reduced at all post-treatment time points (*p* < 0.0001) as shown in Fig. 1-B. The mean subfoveal choroidal thickness (SCT) values at baseline, 1, 2, and 3 months after initiation of faricimab were 215 ± 95 μm, 201 ± 90 μm, 193 ± 88 μm, and 189 ± 82 μm, respectively. SCT was significantly reduced at all post-treatment time points (*p* < 0.0001) as shown in Fig. 1-C. The dry macula rates were 22/62 (35%), 43/62 (69%), and 51/62 (82%) at 1, 2, and 3 months, respectively. Nine eyes did not obtain a dry macula at 3 months after initiation of faricimab, including five eyes with PCV and four eyes with type 1 MNV and/or type 2 MNV. Pigment epithelium detachment (PED) was observed in 34 (54%) eyes at baseline, which was resolved in 8 (24%) eyes and decreased in height in 12 (35%) eyes at 3 months. Polypoidal lesions in patients with PCV showed complete, partial, and no regression in 11 (50%), 4 (18%), and 7 (32%) eyes, respectively, at 3 months. The presence of subretinal fluid (SRF), intraretinal fluid (IRF) and PED are shown in Fig. 2. Retinal pigment epithelium (RPE) tears were observed in two eyes, in one eye, RPE tear occurred after the first faricimab treatment in patients with mixed of type 1 and type 2 (83 y/o male) and in the other eye, it developed after the third faricimab treatment in PCV eye (77 y/o male). There were no other ocular or systemic complications, including arterial thromboembolisms, during this period. Comparison of demographic and clinical characteristics among cases with Type 1 and/or Type 2 macular neovascularisation (MNV), polypoidal choroidal vasculopathy (PCV) and Type 3 (MNV) at baseline and 3 months after initiation of faricimab treatment was summerised in Table 2. The representative cases treated with loading therapy of faricimab are shown in Figs. 3, 4, 5, 6, 7, 8, 9 and 10. A case of RPE tear development was shown in Fig. 11.

**Table 1:** Baseline demographic and clinical characteristics of patients with wet age-related macular degeneration.

	All participants
Number of patients (eyes)	60 (62)
Female, n (%)	20 (33%)
Age (years ± SD)	76 ± 10
Subtype, eyes (%)	
Type1 and/or Type2 MNV	32 (52%)
Type 1 MNV	24
Type 2 MNV	4
Mixed of type 1 and type 2	4
PCV	22 (35%)
Type3 MNV	8 (13%)
BCVA (logMAR ± SD)	0.4 ± 0.42
CFT (μm)	357 ± 165
SCT (μm)	215 ± 95
PED, n (%)	34 (55%)

**Table 2:** Comparison of demographic and clinical characteristics among cases with Type 1 and/or Type 2 macular neovascularisation (MNV), polypoidal choroidal vasculopathy (PCV) and Type 3 (MNV) at baseline and 3 months after initiation of faricimab treatment.

	Total	Type 1 and/or Type 2 MNV	PCV	Type 3 MNV
Number of eyes, n (%)	62	32 (52%)	22(35%)	8 (13%)
Female (%)	20 (33%)	10 (31%)	3 (14%)	7 (100%)
Mean age (years)	76 ± 10	77 ± 9	73 ± 10	84 ± 7
BCVA at baseline (logMAR)	0.40 ± 0.42	0.44 ± 0.50	0.28 ± 0.28	0.56 ± 0.43
BCVA at 3 months (logMAR)	0.32 ± 0.43	0.35* ± 0.49	0.19* ± 0.32	0.51 ± 0.41
Mean CRT at baseline (μm)	357 ± 165	381 ± 183	335 ± 104	321 ± 225
Mean CRT at 3 months (μm)	175 ± 91	195* ± 113	166* ± 52	124 ± 55
Mean SCT at baseline (μm)	215 ± 95	207 ± 97	246 ± 99	164 ± 45
Mean SCT at 3 months (μm)	189 ± 82	176* ± 83	226* ± 84	151 ± 39
Dry macula at 3 months, n (%)	51 (82%)	26 (84%)	18 (82%)	7 (88%)
Complete regression of polypoidal lesion at 3 months, n (%)		–	11 (50%)	–

3 months; at 3 months after initiation of faricimab.

## Discussion

This Japanese multicentre study demonstrated visual and anatomical improvement as well as the general safety of loading therapy with faricimab in untreated eyes with wAMD. BCVA improved 2 months after the first faricimab treatment, which further improved at 3 months. The loading therapy of faricimab gradually induced these improvements in patients with wAMD. In comparison, we reported that the short-term BCVA significantly improved 1 month after the first brolucizumab injection in patients with wAMD, except for those with PCV^10^. Matsumoto et al. reported similar results in patients with type 1 MNV^11^. However, Fukuda et al. reported that visual function significantly recovered 3 months after treatment with aflibercept in eyes with PCV^12^, but no significant recovery was achieved at 3 months by treatment with brolucizumab.

Moreover, 35%, 69%, and 82% of patient eyes in our study attained a dry macula at 1, 2, and 3 months, respectively, and polypoidal lesions completely disappeared in 50% of the patients with PCV. We previously reported that dry macular rates 3 months after brolucizumab treatment were 91%, 88%, and 100% for type 1 and/or type 2 MNV, PCV, and type 3 MNV, respectively^10^. Another study reported dry macula rates of 47.2%, 86.1%, and 94.4% at 1, 2, and 3 months, respectively, after treatment with brolucizumab^11^ in type 1 MNV. Among patients with PCV treated with aflibercept, the SRF-free rates were 44.7%, 76.3%, and 84.2% at 1, 2, and 3 months after aflibercept treatment, respectively, while the corresponding values after brolucizumab treatment were 64.3%, 85.7%, and 100%^12^. Overall, the improvement in visual function and retinal anatomy after treatment with faricimab during loading therapy was comparable to that after treatment with aflibercept. In our cohort, faricimab allowed complete regression of polypoidal lesions in 50% of patients with PCV. In our previous study, the rate of complete regression of polypoidal lesions in eyes treated with aflibercept was 47.8%^13^, which is consistent with other reports^14,15^. In eyes treated with brolucizumab, the regression rate has been reported to be 73.9–78.9%^12,16^. Thus, faricimab and aflibercept have similar effects on the regression of polypoidal lesions.

Furthermore, the loading treatment significantly reduced the SCT in the affected eyes. VEGF is secreted from the basement membrane of the RPE to maintain choroidal homeostasis^17^. Ang-1–Tie 2 receptors are expressed in the choroid^5^, where they maintain the stability of choroidal vessels^18^. As faricimab affects these receptors, it could also affect choroidal vessels. Accordingly, there was a decrease in SCT at 1, 2, and 3 months after initiation of faricimab treatment (by 93%, 90%, and 88%, respectively). In our previous study, the SCT decreased by 91.3%, 89.8%, and 86.5% at 1, 2, and 3 months, respectively, after treatment with aflibercept in PCV cases^19^. Related results regarding the effects of aflibercept ^20,21^ on SCT have been reported. Moreover, in our previous study, SCT in eyes with wAMD decreased by 90.6%, 86.6%, and 84.7% at 1, 2, and 3 months, respectively, after treatment with brolucizumab^22^. In other studies, SCT in eyes with type 1 MNV or PCV decreased by 83.3–89.4%, 82.5–86.0%, and 84.1–84.4% at 1, 2, and 3 months, respectively, after treatment with brolucizumab^11,12^. Faricimab might have a slightly lower impact on the choroid than brolucizumab.

There were two cases of RPE tears: one developed 1 month after the first faricimab treatment in an eye with type 1 and type 2 MNV, while the other developed at 3 months in an eye with PCV. By the end of the loading therapy, the bilateral visual acuity was maintained. In a study conducted by TENAYA and LUCERNE, RPE tears after treatment with faricimab developed in five eyes (1.3%) and three eyes (0.9%), respectively^9^. This trend requires careful monitoring in patients with a large PED, in which active type 1 MNV spreads through sub-RPE fluid^23^.

In the 3 months after initiation of faricimab treatment, no cases with ocular inflammation were observed, even though in the previous phase 3 trials (TENAYA and LUCERNE), 1.3% and 1.5% ocular adverse events were reported in each, respectively, including 0.3% and 0.6% vitritis, 0.5% and 0.6% iritis, and 0.5% and 0.6% uveitis, respectively. Ang-2 may be essential for constructing normal vasculature in response to physiological events ^24^; however, reducing Ang-2 can reduce inflammation, as was shown in a mouse model of myocardial infarction ^25^. It is possible that the anti-inflammatory effects of Ang-2 inhibition contributed to the low rate of ocular inflammation.

The limitations of this study include its retrospective nature and short follow-up period. Thus, further studies are required to verify the long-term efficacy and safety of faricimab in patients with wAMD.

In conclusion, a loading therapy of faricimab effectively improved visual acuity and retinal morphology in eyes with wAMD. Ophthalmologists should carefully monitor safety during follow-up.

## Methods

This study included 63 untreated eyes of 61 patients with neovascular AMD, who visited the Fukushima Medical University Hospital; Kyorin University Hospital; Nihon University Hospital; University of the Ryukyus Hospital; and Tokyo Women’s Medical University Hospital (Japan AMD Research Consortium: JARC) between June 2022 and August 2022. The study protocol was approved by the Institutional Review Board of Fukushima University, Kyorin University Hospital, Nihon University Hospital, Ryukyus Hospital and Tokyo Women’s Medical University Hospital as a retrospective study. All study protocols adhered to the tenets of the Declaration of Helsinki. Written informed consent was obtained from all the patients included in this study. We included untreated patients with wAMD aged ≥ 45 years. We excluded patients with myopia of > − 6 dioptres, a history of uveitis, or a history of vitrectomy. One patient (1 eye) did not return for personal reasons. Accordingly, we included 62 eyes from 60 patients who received faricimab at 1-month intervals for 3 consecutive months, with assessments at baseline and then after 1 and 2 months. The outcomes included visual acuity, fluid resolution rate, CFT, SCT, changes in PED, and polypoidal lesion regression rate in patients with PCV after 3 months. Fluorescein angiography (FA) and indocyanine green angiography (ICGA) were performed using a confocal scanning laser ophthalmoscope (Spectralis HRA + OCT; Heidelberg Engineering) to determine the subtypes of neovascular AMD, including type 1 MNV, type 2 MNV^26^, PCV^27^, and type 3 MNV^26^. PCV was diagnosed based on the presence of polypoidal lesions on ICGA^27^. The diagnosis criteria for type 3 MNV were retinal–retinal anastomosis on early-phase FA or ICGA and a hot spot on late-phase ICGA^28^. We determined the BCVA using the early treatment diabetic retinopathy study (ETDRS) visual acuity chart at Nihon University as well as the Landolt C chart at the other four institutions. BCVA was converted to the logarithm of the minimal angle of resolution (logMAR) units for outcome analyses. On the OCT images, the macula was considered dry if the subretinal and intraretinal fluids were completely resolved. CFT was measured from the superior border of the RPE to the border of the inner retinal layer at the foveal centre. Additionally, SCT was measured as the vertical distance between the hyperreflective line corresponding to Bruch’s membrane under the RPE and the inner scleral boundary at the foveal centre using the caliper function of the OCT (DRI-OCT [Topcon] at the University of the Ryukyus and Tokyo Women’s Medical University; and Heidelberg Spectralis [Heidelberg Engineering Inc.] at Nihon University, Fukushima Medical University, and Kyorin University).

PED was defined as one or more detached areas, the same size as the optic disc, in the macula on FA; its height was also recorded for comparison with previous measurements. The height of PED from the inner layer of Bruch’s membrane to the top of the RPE was measured using OCT. Changes in polypoidal lesions were recorded as complete regression, partial regression, or increase relative to baseline levels as measured using ICGA. Exclusion criteria were massive submacular haemorrhages extending beyond the equator.

### Statistical analysis

Data are presented as the mean ± standard deviation (SD). The Wilcoxon signed-rank test was used to assess changes in visual acuity. One-way analysis of variance was used to compare CRT and SCT before and after treatment. Statistical significance was set at *p* < 0.05. GraphPad Prism version 9 (GraphPad Software, LLC) was used for the statistical analyses.

## Data Availability

The datasets used and/or analysed during the current study available from the corresponding author on reasonable request.
